# Genome-wide association study reveals 14 new SNPs and confirms two structural variants highly associated with the horned/polled phenotype in goats

**DOI:** 10.1186/s12864-021-08089-w

**Published:** 2021-10-28

**Authors:** Jiazhong Guo, Rui Jiang, Ayi Mao, George E. Liu, Siyuan Zhan, Li Li, Tao Zhong, Linjie Wang, Jiaxue Cao, Yu Chen, Guojun Zhang, Hongping Zhang

**Affiliations:** 1 College of Animal Science and Technology, Sichuan Agricultural University, Chengdu, 611130 China; 2 Animal Genomics and Improvement Laboratory, BARC, Agricultural Research Service, USDA, Beltsville, MD 20705 USA; 3 Nanjiang Yellow Goat Scientific Research Institute, Bazhong, 635600 China

**Keywords:** Goat, Whole-genome sequencing, GWAS, Horn, SNP, CNV, Genetic testing

## Abstract

**Background:**

There is a long-term interest in investigating the genetic basis of the horned/polled phenotype in domestic goats. Here, we report a genome-wide association study (GWAS) to detect the genetic loci affecting the polled phenotype in goats.

**Results:**

We obtained a total of 13,980,209 biallelic SNPs, using the genotyping-by-sequencing data from 45 Jintang Black (JT) goats, which included 32 female and nine male goats, and four individuals with the polled intersex syndrome (PIS). Using a mixed-model based GWAS, we identified two association signals, which were located at 150,334,857–150,817,260 bp (*P* = 5.15 × 10^− 119^) and 128,286,704–131,306,537 bp (*P* = 2.74 × 10^− 15^) on chromosome 1. The genotype distributions of the 14 most significantly associated SNPs were completely correlated with horn status in goats, based on the whole-genome sequencing (WGS) data from JT and two other Chinese horned breeds. However, variant annotation suggested that none of the detected SNPs within the associated regions were plausible causal mutations. Via additional read-depth analyses and visual inspections of WGS data, we found a 10.1-kb deletion (CHI1:g. 129424781_129434939del) and a 480-kb duplication (CHI1:150,334,286–150,818,098 bp) encompassing two genes *KCNJ15* and *ERG* in the associated regions of polled and PIS-affected goats. Notably, the 10.1-kb deletion also served as the insertion site for the 480-kb duplication, as validated by PCR and Sanger sequencing. Our WGS genotyping showed that all horned goats were homozygous for the reference alleles without either the structural variants (SVs), whereas the PIS-affected goats were homozygous for both the SVs. We also demonstrated that horned, polled, and PIS-affected individuals among 333 goats from JT and three other Chinese horned breeds can be accurately classified via PCR amplification and agarose gel electrophoresis of two fragments in both SVs.

**Conclusion:**

Our results revealed that two genomic regions on chromosome 1 are major loci affecting the polled phenotypes in goats. We provided a diagnostic PCR to accurately classify horned, polled, and PIS-affected goats, which will enable a reliable genetic test for the early-in-life prediction of horn status in goats.

**Supplementary Information:**

The online version contains supplementary material available at 10.1186/s12864-021-08089-w.

## Introduction

As a major form of sexual weaponry in intra-male competition, horns in natural populations of ruminants also serve the purpose of self-defense against predators [1]. In modern livestock husbandry, however, horned animals are less desirable because they pose hazards in herds and cause more difficulties in management and transportation, thereby increasing the economic cost. Dehorning can be achieved via the removal of horn buds for animals in early life (e.g., calves [2, 3] and goat kids [4, 5]) through heat cauterization, caustic agents, and surgical methods. Nevertheless, the physical disbudding causes pain and distress to young animals, which violates animal welfare [6, 7]. Therefore, there is a long-term interest to breed polled animals by genetic selection in the modern cattle (e.g., Holstein [8]), sheep (e.g., Polled Dorset), and goat (e.g., Matou goat, a polled Chinese breed) industries.

To date, the genetic basis of the horned and polled phenotypes has been extensively investigated in ruminants. Although their genomic positions are closely located on the proximal end of bovine chromosome 1, a total of four different genetic variants are associated with the polled phenotypes in cattle with diverse origins [9]. As well-known, *RXFP2* is a major candidate gene influencing the horned and polled phenotypes [10–12] and even horn numbers in sheep [13], whereas the causal mutations seem to be not the same in different breeds [14, 15]. In goats, several SNPs residing at ~ 129 Mb on chromosome 1 were genome-wide significantly associated with the polled phenotype in three Australian breeds [16]. However, the low density of SNPs from the Goat SNP50 chip could not provide detailed clues for the in-depth examination of the genetic architecture. Furthermore, an 11.7-kb deletion located at ~ 129 Mb on chromosome 1 is thought to be responsible for the polled intersex syndrome (PIS) in Saanen goats [17], which adds to the complexity of the identification of the causal variants affecting the polled phenotype in goats. A recent study using the long-read sequencing finely mapped the length of the PIS deletion from 11.7-kb to be 10,159 bp in Saanen goats [18]. More interestingly, a ~ 480-kb duplicated sequence originally residing at the position of 150,334,286–150,818,098 bp on chromosome 1 inversely inserted into the PIS position, which is also the case for Chinese PIS goats [19]. Based on a diagnostic PCR assay, Simon et al. further reported that normal polled goats are heterozygous for the PIS deletion [18]. However, there is an absence of a genome-wide mapping study that could provide strong evidence of the correlation between structural variants and horn status. Taken together, the molecular genetic mechanism underlying the polled phenotype remains elusive in domestic goats.

Compared to low-density SNP chips, millions of SNPs detected from whole-genome sequencing (WGS) data permit fine mapping of association signals in genome-wide association studies (GWAS), which make it more feasible to find candidate genes affecting complex traits [20] and even causal mutations underlying Mendelian traits [21] in livestock. In this study, we performed a GWAS to detect the genetic loci affecting the polled phenotype in goats by using the genotyping-by-sequencing data from 45 Jintang Black (JT) goats. Based on the sequence characteristics of the associated genomic region, we further demonstrated the usefulness of a diagnostic PCR with two primer pairs for the classification of horned, polled, and PIS-affected phenotypes in several Chinese goat breeds, which provide a robust molecular genetic test for the early-in-life prediction of horn status in goats.

## Results

The alignment of short-read WGS data against the goat reference genome (i.e., the ARS1 assembly) yielded an average sequencing depth of 6.24× (ranging from 5.52× to 7.28×) and genome coverage of 98.80% over 45 JT goats (Additional file 1). A total of 14,112,599 single nucleotide polymorphisms (SNPs) (14,047,290 biallelic and 65,309 multiallelic) and 1,303,926 short insertions and deletions (Indels) were identified across the goat autosomal genome.

The individuals for the GWAS mapping were randomly selected from one JT population. A quick principal component analysis (PCA) showed two sub-groups among the total of 45 animals, possibly due to familial relatedness. However, the total fraction of genetic variance explained by the first two principal components was low (12.01 and 1.68%) (Additional file 2).

### Two genomic regions both on chromosome 1 were associated with the horned and polled phenotypes

**Fig. 1:**
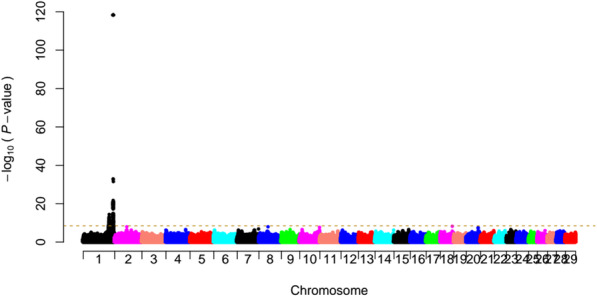
Genome-wide association study for the polled phenotype in JT goats based on a linear mixed model. A mixed-model based GWAS for the polled phenotype in 45 JT goats that included 32 female, nine male, and four PIS-affected individuals. Manhattan plot of the association of 13,980,209 biallelic SNPs on autosomes 1–29 with the polled phenotype. The chromosomes are plotted separately by color. The horizontal dashed line indicates the genome-wide Bonferroni-corrected significance level

After the Hardy-Weinberg test (Fisher’s exact test) in PLINK, 13,980,209 biallelic SNPs (*P*-value > 10^− 6^) on all 29 autosomes remained for the GWAS. We carried out a mixed-model association analysis using all 45 goats, including 32 JT female goats plus additional nine male goats and four PIS cases, which corrected for the population structure. We identified two association signals that consisted of 528 significant SNPs (F-test, Bonferroni-corrected *P* < 0.05, −log_10_ *P* = 8.45, the genomic inflation factor λ = 1.04) that were all located on chromosome 1 (Fig.1 and Additional file 3). The strongest association peak spanned 150,334,857–150,817,260 bp (i.e., ~ 482.40 kb in length) on chromosome 1 and was composed of 455 genome-wide significant SNPs with 14 most associated SNPs (*P* = 5.15 × 10^− 119^) at 150,363,2033–150,734,900 bp. The second association signal was located at 128,286,704–131,306,537 bp on chromosome 1, which included 73 genome-wide significant SNPs with the leading one at 130,095,365 bp (*P* = 2.74 × 10^− 15^).

### Characterization of SNPs and Indels within the two association signals

**Fig. 2:**
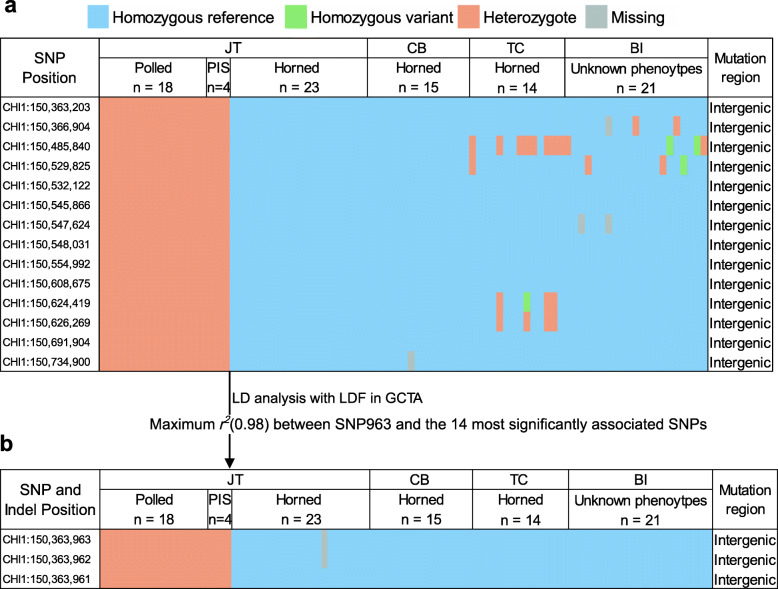
Summary of the genotype distributions of the 14 genome-wide most significantly associated SNPs and the loci showing highest LD with them. **a** The genotype distributions of the 14 most significantly associated SNPs in three domestic goats breeds (i.e., JT, CB, and TC) and Bezoars identified by WGS. **b** The genotype distributions of one SNP showed the highest LD with the 14 most associated SNPs, and its adjacent two Indels in JT, CB, TC, and Bezoars identified by WGS

According to variant annotation, the 14 genome-wide most significantly associated SNPs all resided in intergenic regions (Fig. 2a). Considering the genotypes at these loci, all 23 horned animals were homozygous and carried the reference alleles (the ARS1 reference genome is assembled using genomic DNA of a horned San Clemente goat), whereas the 18 polled animals and four PIS-affected goats were heterozygous (Fig. 2a), regardless of sex. In other words, the genotype distributions at these loci were completely correlated with the horned and polled phenotypes in the 45 JT goats. The genotypes obtained from WGS data also showed that the 14 SNP loci were almost fixed for the reference alleles in 15 Chengdu Brown (CB) goats and 14 Tibetan Cashmere (TC) goats that are all horned animals (Fig. 2a), which were the same for 21 Bezoar ibexes (*Capra aegagrus*). The occurrence of heterozygous and homozygous variant genotypes at several sites was possibly due to breed differences. Although we did not know the phenotypes of the 21 Bezoars, these results suggested that the reference alleles at these loci were ancestral, while the variant ones were derived alleles.

A total of 4389 biallelic and 37 multiallelic SNPs, as well as 383 Indels, were detected in the strongest associated region that encompasses two genes, *KCNJ15* (*potassium inwardly rectifying channel subfamily J member 15*) and *ERG* (*ETS transcription factor ERG*) (Additional file 4). To determine the causal variants responsible for the polled phenotype, we first examined whether any SNPs with functional impacts exist within both genes. Although the variant annotation identified a stop-gained C > G mutation (i.e.,CHI1: 150,634,326, UAC to UAG, p.Tyr30*) within *ERG* in seven goats, PCR amplification and Sanger sequencing showed that this mutation did not exist in seven goats and the in silico prediction was false positive (Additional file 5). Two missense SNPs with moderate impacts were also detected in *KCNJ15* (CHI1: 150,485,599, c.74 T > C, p.Ile25Thr) and *ERG* (CHI1: 150,561,787, c.1070G > C, p.Arg357Pro), but they did not yet show significant associations.

Among the 4389 biallelic SNPs, one biallelic SNP (G > T) at 150,363,963 bp (hereafter referred to as the SNP963 for simplicity) on chromosome 1 showed the strongest linkage disequilibrium (LD, *r*^*2*^ = 0.98) with the 14 most associated SNPs (Fig. 2b). The SNP963 was significantly associated with the polled phenotype (*P* = 1.93 × 10^− 33^), and both adjacent insertions (i.e., insTTT at 150,363,961 bp and insT at 150,363,962 bp) were also found at the genomic positions 2 bp before the SNP963. Interestingly, the three sites were phased based on the variant calling using GATK, which resulted in that the reference haplotype ‘ACG’ changed into the mutant haplotype ‘ATTTCTT’. All 52 horned goats (i.e., 23 JT, 15 CB, and 14 TC goats) and 21 Bezoars were homozygous for the three sites except for one goat with the missing genotype at the SNP963 locus, whereas all 18 polled goats and four PIS-affected cases were heterozygous based on the genotypes from WGS data (Fig. 2b).

A total of 14,854 biallelic and 44 multiallelic SNPs, and 1355 Indels, were identified in the second association peak (i.e., 128,286,704–131,306,537 bp on chromosome 1). According to variant annotation, the leading associated SNP (130,095,365 bp) resided in intergenic regions, and it showed the highest LD (*r*^*2*^ = 0.82) with an intergenic SNP at 129,548,618 bp. Furthermore, this associated region harbors 27 genes (21 protein-coding genes and 6 RNA genes), such as *NMNAT3*, *CRBP1*, *PIK3CB*, *SOX14*, and *FOXL2* (ENSCHIG00000026523) (Additional file 4). The variant annotation analysis predicted 25 SNPs with moderate impacts within eight genes in this associated region, but they did not reach the genome-wide significance.

### Identification and characterization of structural variants in the two associated regions

**Fig. 3:**
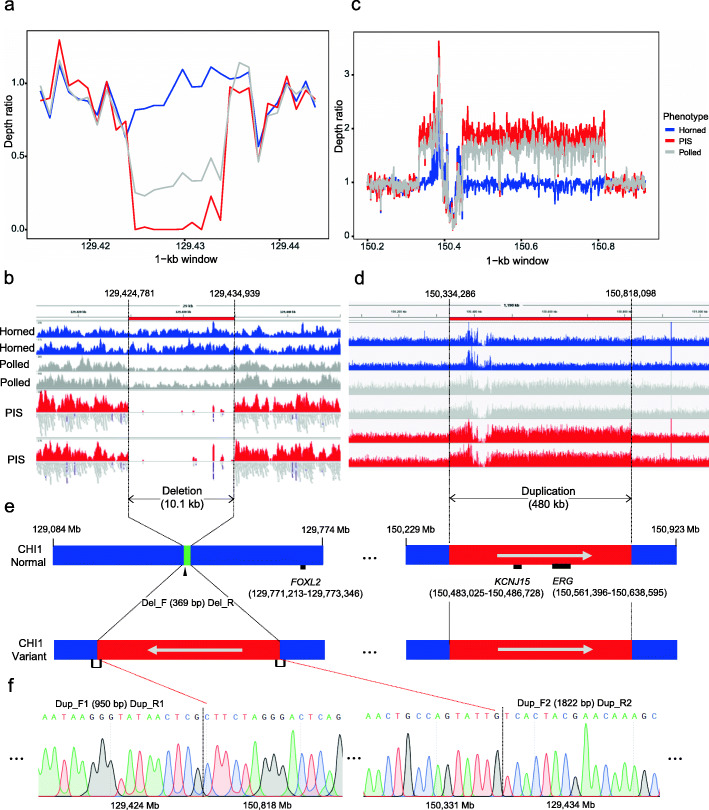
Characterization of two structural variants in the two associated regions. **a** Genomic coverage of different phenotype groups at the 10.1-kb deletion site (adjusted for the chromosome-wide coverage and calculated in 1-kb sliding windows). **b** The screen capture of aligned short reads featuring a 10.1 kb deletion between 129,424,781 and 129,434,939 bp on chromosome 1 using IGV. **c** Genomic coverage of different phenotype groups at the 480-kb duplication site (adjusted for the chromosome-wide coverage and calculated in 1-kb sliding windows). **d** The screen capture of aligned short reads featuring a 10.1 kb deletion between 150,334,286 and 150,818,098 bp on chromosome 1 using IGV. **e** A diagram showed the inverse insertion of the 480-kb duplication at the PIS location in the genomes of polled and PIS goats. **f** Confirmation of the inverse insertion of the 480-kb duplication into the PIS deletion in PIS goats by PCR amplification and Sanger sequencing

To further ascertain the causative variations underlying the two association signals, we sought to identify structural variants (SVs) within or around the two association peaks using WGS data from 74 domestic goats and 21 Bezoars. The read-depth analysis in 1-kb sliding windows first detected a near 10-kb deletion (129,424,001–129,433,500 bp) on chromosome 1 in polled and PIS-affected goats (Fig. 3a and Additional file 6). Visual inspection of WGS data using Integrative Genomics Viewer (IGV) showed that the breakpoints occurred at 129,424,781 and 129,434,939 bp (i.e.,CHI1:g. 129424781_129434939del), which resulted in the exact length of 10,159 bp for this deletion (Fig. 3b). Based on WGS genotyping using CNVcaller, all 52 horned animals (i.e., 23 JT, 15 CB, and 14 TC goats) and 21 Bezoars were homozygous for the reference allele without the deletion of 10.1-kb sequence, whereas 12 out of 18 polled goats were heterozygous (Table 1 and Additional file 6). The remaining six polled goats and four PIS goats were homozygous for this deletion.

**Table 1:** Genotype Distributions of both SVs in three Chinese goat breeds and Bezoars analyzed by WGS and their PCR validations

Breed	Phenotype	Sample size	Genotype of the 10.1-kb deletion			Genotype of the 480-kb duplication			Two PCR products^a^	
			Del/Del	Ref/Del	Ref/Ref	Dup/Dup	Ref/Dup	Ref/Ref	Deletion	Duplication
JT	polled	18	6	12	0	7	11	0	P (*n* = 18)	P (*n* = 18)
JT	PIS	4	4	0	0	4	0	0	P (*n* = 4)	P (*n* = 4)
JT	horned	23	0	0	23	0	0	23	A (*n* = 23)	A (*n* = 23)
CB	horned	15	0	0	15	0	0	15	A (*n* = 15)	A (*n* = 15)
TC	horned	14	0	0	14	0	0	14	A (*n* = 14)	A (*n* = 14)
Bezoars	unknown	21	0	0	21	0	0	21	–	–

*Note*: ^a^ The two PCR products in the deletion and duplication regions were 369 bp and 1822 bp, respectively. The letter “P” means the presence of a PCR product, and the letter “A” means the absence. The WGS data from 21 Bezoars were downloaded from NCBI, and therefore their genomic DNA was unavailable

The read-depth analysis in 1-kb sliding windows also revealed four adjacent duplicated sequences (i.e., 150,334,001–150,378,000 bp, 150,379,501–150,446,000 bp, 150,447,001–150,540,500 bp, and 150,545,001–150,818,500 bp) that overlapped with the most associated region in polled and PIS-affected goats (Fig. 3c and Additional file 6). We then merged and determined that the duplicated sequence was 483,813 bp in length and located between 150,334,286 and 150,818,098 bp, based on the alignment information of several discordant read pairs in IGV (Fig. 3d). The WGS genotyping calls showed that the genotype distributions of the four duplications, except for the segment spanning 150,379,501–150,446,000 bp, were almost the same as those of the 10.1-kb deletion across the three phenotype groups. Specifically, all 52 horned animals and 21 Bezoars were homozygous for the reference allele without this duplication, whereas 11 out of 18 polled goats were heterozygous (Table 1 and Additional file 6). The remaining seven polled goats and four PIS goats were homozygous for this duplication. The genome annotation showed that the duplicated sequence only includes two genes, *KCNJ15* (150,483,025–150,486,728 bp on the forward strand) and *ERG* (150,561,396–150,638,595 bp on the minus strand) (Fig. 3e). Furthermore, several discordant read pairs in IGV and Sanger sequencing revealed that the duplicated sequence was inversely inserted into the genomic position of the 10.1-kb deletion (Fig. 3e and f).

**Fig. 4:**
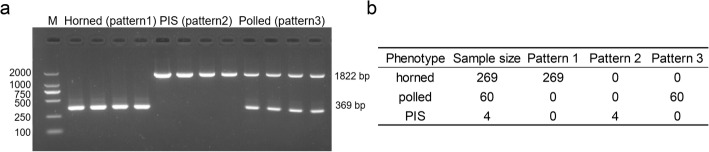
Validation of two structural variants in 333 animals from four Chinese goat breeds by PCR amplification and agarose gel electrophoresis. **a** Agarose gel picture of the two PCR products (i.e., the 369-bp and 1822-bp fragments) for four horned, four polled, and four PIS-affected goats. Pattern 1 represented that the presence of only the 369-bp fragment; Pattern 2 represented the presence of only the 1822-bp fragment; the pattern 3 represented the presence of both the 369-bp and 1822-bp fragments. **b** Summary of the validation of two SVs in 333 samples from four Chinese goat breeds (i.e., JT, CB, TC, and NJ). Note: the total 333 samples included the animals with the WGS data, except for Bezoars

We further carried out PCR amplification to validate the 10.1-kb deletion and the 480-kb duplication in 333 goats from JT and three other Chinese horned goat breeds (CB: *n* = 23, TC: *n* = 42, Nanjiang Yellow goats - NJ: *n* = 118), which included the 74 goats that were whole-genome sequenced. The PCR results for both SVs showed, as expected, only the 369-bp segment was present (i.e., Pattern 1) in a total of 269 horned goats from four Chinese breeds, whereas only the 1822-bp fragment was present (i.e., Pattern 2) in four PIS-affected goats (Fig. 4a and b, Additional file 7), implying that the complex structural variant did not occur in the genomes of horned goats. Both the fragments were present (i.e., Pattern 3) in 60 polled goats from JT, which suggests the above homozygous genotypes for both the deletion and duplication using WGS data were less reliable for several polled goats (six deletions and seven duplications, see Additional file 6). These discrepancies could be due to unevenness of WGS or SNP impacts on mapping efficiency. In summary, our results indicated that the presence or absence of the two-fragment combination could accurately classify horned, polled, and PIS-affected goats.

## Discussion

In this study, we conducted a GWAS to identify the genetic loci affecting the polled phenotype in goats using a high-density SNP map derived from WGS. Strikingly, all genome-wide significant SNPs were located on chromosome 1 and clustered into two association peaks, of which the strongest signal was between 150,334,857 and 150,817,260 bp. The second association peak spanned 128,286,704–131,306,537 bp and was in line with a previous GWAS for the polled phenotype using the goat SNP50 chip [16]. Interestingly, the second associated region also overlapped with the genetic locus responsible for the PIS in goats [17, 18], which made it more challenging to identify the causal variants affecting the polled phenotype in goats.

Regardless of sex, all polled goats included in this work were heterozygous for the 14 most significantly associated SNPs. By contrast, these loci were almost fixed for the reference alleles of horned goats from JT and two other Chinese goat breeds based on WGS data. In other words, the genotype distributions at the 14 most significantly associated SNP loci showed complete correlations with horn status, in agreement with the dominant Mendelian inheritance of the polled phenotype in goats. Therefore, these SNPs can be regarded as potential indicators of the presence or absence of horns in goats. However, the variant annotation analysis showed that these SNPs resided at intergenic regions of the goat genome.

The two associated genomic regions encompass several genes, including *ESYT3*, *SOX14*, *FOXL2*, *ERG*, and *KCNJ15*. Although we found several SNPs with moderate functional impacts within these genes through in silico predictions, they did not show significant associations. Of these genes, both *ESYT3* and *SOX14* were differentially expressed between fetal sheep horn bud and nearby skin, but their abundances were relatively low (in FPKM unit) in horn buds [22]. *FOXL2* is a well-known female sex-determining gene in mammals, supported by the studies using gene knockout technology in mice [23] and goats [24]. Notably, the analyses of 221 transcriptomes from bovids and cervids revealed that *FOXL2* was one of the two headgear-specific genes [22], suggesting its potential roles in horn growth. A previous study has demonstrated that TMPRSS2:ERG can directly regulate the expressions of three osteoblastic markers (i.e., *ALPL*, *COL1A1*, and *ET-1*) at the cell level [25], indicating that ERG is involved in bone metabolism. The expression of *KCNJ15* increases in otosclerosis patients [26], which implies this gene is directly involved in abnormal bone remodeling in humans. In livestock, the SNPs within or around the *ERG* gene were significantly associated with multiple traits (e.g., feet and leg conformation, stature, and stillbirth) in Holstein cattle [27]. These findings support the potential functions of these genes in regulating horn growth and metabolism. However, variant annotations and the GWAS demonstrate that none of the SNPs and Indels within these genes were plausible causal variants affecting the polled phenotype in goats. Interestingly, four different genetic variants controlling the polled phenotype all are structural variants in cattle [9]. As discussed below, these results prompted us to investigate non-coding regulatory elements or structural variants in the associated regions of the goat genome.

In the associated genomic regions in polled and PIS goats, we also detected two SVs (i.e., the PIS deletion located at 129,424,781–129,434,939 bp and a 480-kb duplicated sequence originally from 150,334,286–150,818,098 bp on chromosome 1), while both variants did not exist in the genomes of horned goats and Bezoars. Here, the PIS deletion shortened from a previously known length of 11.7-kb [17] to 10.1-kb, which was consistent with two WGS-based studies [18, 19] and demonstrated that the strength of WGS data in the identification of structural variations. According to WGS genotyping, all horned goats from three Chinese breeds and Bezoars were homozygous for the reference alleles without the deletion and the duplication, whereas the PIS-affected goats were homozygous for the deletion and the duplication, which was consistent with previous findings [18, 19] and our PCR results. Thus, we concluded that all normal polled goats were heterozygous for both the SVs, supported by the findings in 23 goat breeds mainly from Europe [18].

Consistent with previous studies [18, 19], our results demonstrated that the 480-kb duplicated segment, completely harboring the genes *KCNJ15* and *ERG*, was inversely inserted at the location of the PIS deletion. Given the potential functions of *KCNJ15*, *ERG*, and *FOXL2*, there might be interactions among these genes, or the dosage effects of *KCNJ15* and *ERG* might exist due to the duplication. However, the comparison of the 3D structures of the PIS variant between intersex and normal goats did not provide strong evidence of how the sequences interact [19]. In summary, we identified the major loci in this study, although the molecular genetic basis of the polled phenotypes remains unclear.

There is a long-term interest in finding the molecular genetic markers (e.g., SNPs and microsatellites) for the accurate prediction of polled and horned phenotypes in ruminants. For example, a recent study demonstrated that an optimized genetic test containing five biallelic SNPs successfully predicted a genotype in 99.96% of 20,636 animals from multiple cattle breeds (e.g., Angus, Charolais, and Holstein) [28]. Although the highest prediction accuracy of 0.725 was present in males based on the phenotype classification of horned vs. non-horned, there were differences in the prediction accuracy between males and females using different statistical models and phenotype classification scenarios [29]. As mentioned above, the 14 most significantly associated SNPs identified in our work provided potential indicators for horn status in goats. Furthermore, a diagnostic PCR using two primer pairs in both SVs (i.e., a 10.1-kb deletion and a 480-kb duplication) enabled us to classify horned, normal polled, and PIS goats in several Chinese goat breeds, which provided a simple and general genetic test from the view of the application.

## Conclusion

In summary, our results demonstrated that two genomic regions located at the distal end of chromosome 1 are highly associated with the horned and polled phenotypes in goats. A diagnostic PCR enabled us to accurately classify horned, polled, and PIS-affected goats, which provided a reliable genetic test for the early-in-life prediction of horn status in goats.

## Methods

### Animals and whole-genome sequencing

In this study, 32 female Jintang Black goats (JT) were randomly sampled from a breeding farm in Jintang County, Sichuan Province, China, including 17 horned and 15 polled animals. Additionally, four polled JT individuals with PIS were included. According to the PCR amplification of a 272-bp fragment within the SRY gene, the four PIS-affected goats were karyotypically XX individuals (Additional file 8 and see Additional file 9 for the primer information). Genomic DNA was extracted from blood using TIANmap Genomic DNA Kit (TIANGEN BIOTECH, China). The integrity and yield of genomic DNA were assessed and verified using agarose gel electrophoresis and a Nanodrop spectrophotometer (Thermo Fisher Scientific, Waltham, MA), respectively. The DNA samples were then sequenced with the paired-end 150 bp mode on an Illumina NovaSeq 6000 sequencer at Novogene (Beijing, China).

We also used short-read WGS data for nine male JT goats (six horned and three polled), 15 male Chengdu Brown goats (CB, a horned breed), and 14 Tibetan Cashmere goats from Coqen (TC, a horned breed) that was generated in our previous work [30] and have been deposited in NCBI (dataset number: PRJNA548681, https://www.ncbi.nlm.nih.gov/sra/?term=PRJNA548681). Moreover, we included WGS data for 21 Bezoar ibexes, which were downloaded from NCBI (dataset number: PRJEB3136, https://www.ncbi.nlm.nih.gov/sra/?term=PRJEB3136) as reported in our previous work [30]. In brief, WGS data for a total of 45 JT goats can be used for GWAS in this work, whereas WGS data from CB and TC breeds as well as Bezoar ibexes were mainly used in the further analyses of the variants in the associated regions identified by GWAS.

### Short-read alignment and variant calling and annotation

After filtering the read pairs containing adapter sequence, we conducted quality control of the raw reads using Trimmomatic [31] (v0.36), with the following parameters: EADING:20, TRAILING:20, SLIDINGWINDOW:4:20, and MINLEN:50. The high-quality reads were then mapped against the goat reference genome [32] (assembly ARS1, https://asia.ensembl.org/index.htm) using the ‘mem’ algorithm of BWA [33] (v0.7.12). Picard software (v2.10.6) (http://broadinstitute.github.io/picard/) was applied to remove the duplicated reads followed by local realignment around existing Indels and base quality score recalibration using GATK [34] (v3.8–0). The ARS1 goat assembly was generated from a horned adult male San Clemente goat.

To obtain quality variants, we performed filtering of the raw variant calls (SNPs and Indels) using GATK with the following cut-offs: QUAL < 100.0, QD < 2.0, MQ < 40.0, FS > 60.0, MQRankSum <− 12.5, ReadPosRankSum < − 8.0 and DP < 100. The highest-confidence variant sites were then obtained after discarding the variants with minor allele frequency (MAF) < 0.05 and missing genotype > 10% at the meta-population/population level, using VCFtools [35]. The biallelic SNPs were finally extracted and were used for the subsequent analyses. In addition, SnpEff [36] (v4.3) was also used for variant annotation and effect prediction of SNPs. SnpEff provides a simple assessment of the putatively functional impacts of variants (e.g., HIGH, MODERATE, or LOW impact) for protein-coding genes.

We also employed CNVcaller [37] (a read-depth approach) to detect and genotype copy number variation regions (CNVRs) across autosomes. To calculate the read-depth signal, we first divided the reference genome into 1-kb overlapping sliding windows, which was recommended for the WGS data with sequencing depth of < 10×. We detected raw CNVRs and merged them into final CNVRs with the parameters ‘-f 0.1 -h 1 -r 0.5’. The parameter ‘-r 0.5’ means that raw adjacent CNVRs were merged into one CNVR if the correlation coefficient of their RDs were ≥ 0.5. In addition, the alignment results of WGS data were visualized with Integrative Genomics Viewer (IGV) [38] to confirm the existence of several CNV loci of interest.

### Population genetics analysis and genome-wide association study

The VCFtools program [35] was applied to convert the variant data file from VCF format to PLINK format [39]. To examine whether the population structure existed in the sampled population, we performed the principal component analysis (PCA) using GCTA [40] (v1.26.0).

We performed single-maker GWAS to identify the loci of interest using a linear mixed model implemented in EMMAX (F-test) [41], given that the population structure existed in the analyzed data. The genomic inflation factor (i.e., λ) of the test statistics was calculated as the slope of a linear regression between observed and theoretical quantiles in R [42] (version 3.6.0). Manhattan plots were drawn with a custom R script. For the LD analysis among SNPs, the LDF function with default parameters (−-ld-wind 5000 --ld-sig 0.05) in GCTA [40, 43], which uses the simple regression approach instead of a classical formula, was applied to find SNPs that are in significant LD with the target SNPs.

### PCR and sanger sequencing of one SNP and two structural variants

Given its potential biological implications, we first conducted PCR and Sanger sequencing to validate a stop-gained C > G mutation (i.e., CHI1:150634326, UAC to UAG, p.Tyr30*) detected from the short-read sequencing data. A primer pair (Additional file 9) was designed to amplify a 574-bp target fragment that contained this SNP. The PCR was performed using the genomic DNA of the seven goats that carry the mutant allele (by the in silico prediction) as a template. The PCR product from each animal was then sequenced on an ABI 3730XL sequencer (Applied Bio-System, USA) at Tsingke Biological Biotechnology Co., Ltd. (Chengdu, China) using the forward primer. The target sequences were then analyzed with the DNASTAR software (v.7.1).

For the 10.1-kb deletion and the 480-kb duplication identified using WGS data, we carried out the PCR amplification to verify the presence of both the SVs using two primer pairs (Additional file 9). The Del_F and Del_R primer pair amplify a 369-bp fragment when the 10.1-kb sequence is present; Dup_F and Dup_R amplify an 1822-bp fragment when the 480-kb duplication is present and inversely inserts at the location of the 10.1-kb deletion. We then used the combination of the 1822-bp and 369-bp fragments to classify horned, polled, and PIS-affected goats, in 333 sampled goats from four Chinese goat breeds (i.e., JT: *n* = 150 [86 horned, 60 polled, and 4 PIS-affected], CB: *n* = 23, TC: *n* = 42, and NJ: *n* = 118 [54 male, 64 female]). The PCR products were resolved by agarose gel electrophoresis.

## Supplementary Information

**Additional file 1: Table S1.** Summary of mapping statistics for 45 JT goats in this study.

**Additional file 2: Fig. S1.** PCA of the 45 sampled goats based on the identified biallelic SNPs. The red, green, and blue circles represented female, male, and PIS goats, respectively.

**Additional file 3: Table S2.** Lists of the genome-wide significantly associated SNPs for the polled phenotype in 45 JT goats.

**Additional file 4: Table S3.** Lists of genes within the two association signals.

**Additional file 5: Fig. S2.** Validation of a mutation within the *ERG* gene through PCR and Sanger sequencing.

**Additional file 6: Table S4.** Genotypes and normalized copy numbers of both SVs in 74 domestic goats and 21 Bezoars analyzed by WGS.

**Additional file 7: Table S5.** The detailed information of validation of both SVs in 333 animals from four Chinese goat breeds.

**Additional file 8: Fig. S3.** The validation of genetic sex for four PIS goats by PCR amplification and agarose gel electrophoresis.

**Additional file 9: Table S6.** The information of PCR primer pairs used in this study.

## Data Availability

The newly generated raw sequencing data for 36 Jintang Black goats (32 female goats and four PIS animals) for GWAS in this study are available from the NCBI SRA database (accession number: PRJNA734084, https://www.ncbi.nlm.nih.gov/bioproject/PRJNA734084) and were provided in Table S1.

## Abbreviations

BI: Bezoar ibex
CB: Chengdu Brown goats
NJ: Nanjiang Yellow goats
JT: Jintang Black goats
TC: Tibetan Cashmere goats
WGS: Whole-genome sequencing
SNP: Single nucleotide polymorphism
Indels: Short insertions and deletions
CNV: Copy number variation
CNVR: CNV region
MAF: Minor allele frequency
CHI: *Capra hircus* chromosome
