# Correction to: Genome-wide association study reveals 14 new SNPs and confirms two structural variants highly associated with the horned/polled phenotype in goats

**DOI:** 10.1186/s12864-022-08361-7

**Published:** 2022-02-10

**Authors:** Jiazhong Guo, Rui Jiang, Ayi Mao, George E. Liu, Siyuan Zhan, Li Li, Tao Zhong, Linjie Wang, Jiaxue Cao, Yu Chen, Guojun Zhang, Hongping Zhang

**Affiliations:** 1 College of Animal Science and Technology, Sichuan Agricultural University, Chengdu, 611130 China; 2 Animal Genomics and Improvement Laboratory, BARC, Agricultural Research Service, USDA, Beltsville, MD 20705 USA; 3 Nanjiang Yellow Goat Scientific Research Institute, Bazhong, 635600 China

**Correction to**: **BMC Genomics 22, 769 (2021)**

**https://doi.org/10.1186/s12864-021-08089-w**

Following publication of the original article [1], the authors noted several typographical errors [corrections in boldface]:

1. The second sentence of the first paragraph of the Results should read: “A total of **14,112,599** single nucleotide polymorphisms (SNPs) (**14,047,290** biallelic and **65,309** multiallelic) and **1,303,926** short insertions and deletions (Indels) were identified across the goat autosomal genome.”

2. The last sentence of the first paragraph of the ‘Short-read alignment and variant calling annotation’ should read: “The **ARS1** goat assembly was generated from a horned adult male San Clemente goat.”
3. The second-to-last sentence of the last paragraph of the ‘Methods’ section should read: “We then used the combination of the 1822-bp and **369-bp** fragments to classify horned, polled, and PIS-affected goats, in 333 sampled goats from four Chinese goat breeds (i.e., JT: *n* = 150 [86 horned, 60 polled, and 4 PIS-affected], CB: *n* = 23, TC: *n* = 42, and NJ: *n* = 118 [54 male, 64 female]).”

The authors apologize for any inconvenience that these errors may have caused.

The original article [1] has been updated.
