# Supplementary figures and images for: Genome-wide association study reveals 14 new SNPs and confirms two structural variants highly associated with the horned/polled phenotype in goats
Source: BMC Genomics. 2021 Oct 28;22:769. doi: 10.1186/s12864-021-08089-w (PMC8555091; doi:10.1186/s12864-021-08089-w)

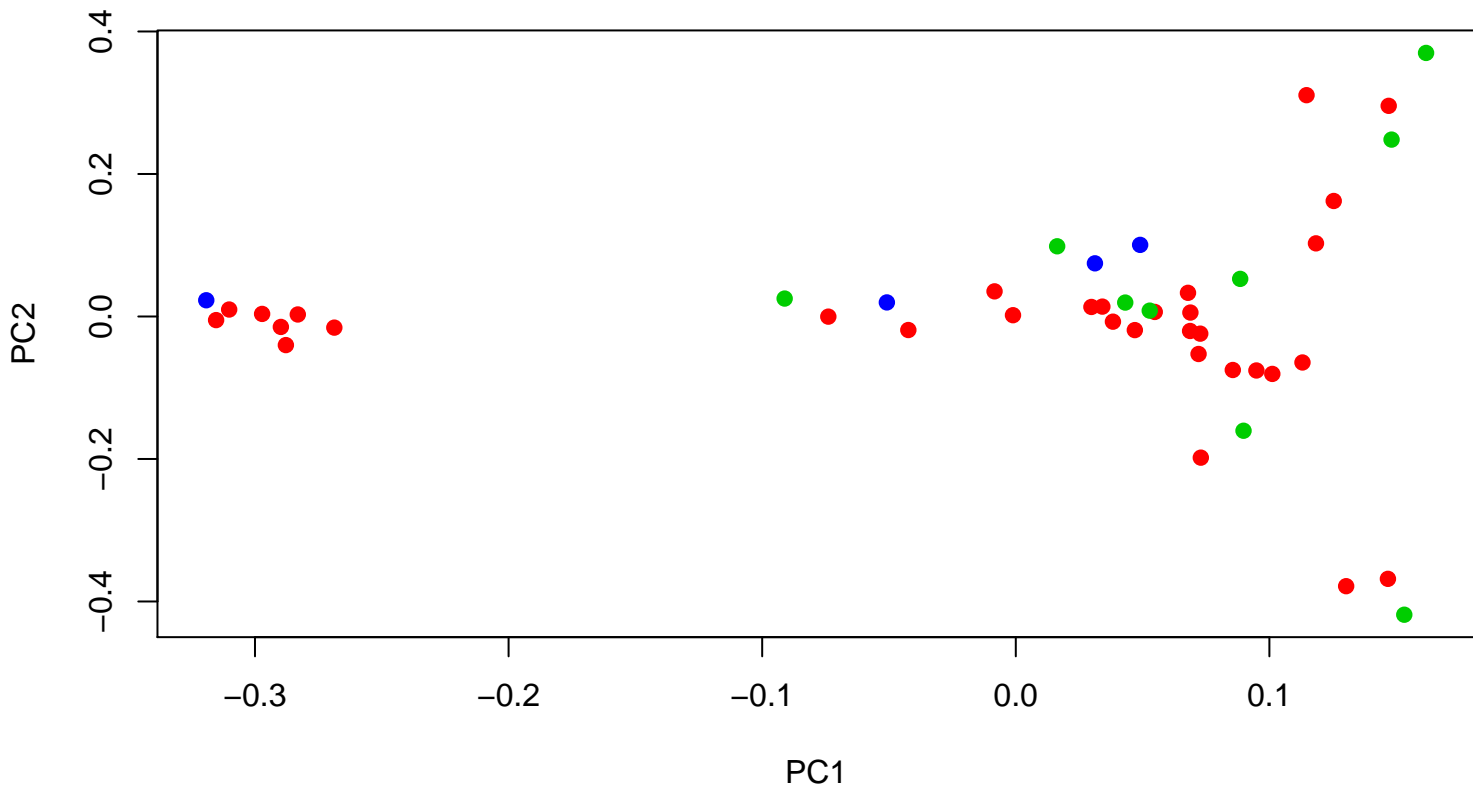

Supplement: Supplementary file 2 — Additional file 2: Fig. S1. PCA of the 45 sampled goats based on the identified biallelic SNPs. The red, green, and blue circles represented female, male, and PIS goats, respectively. [file 12864_2021_8089_MOESM2_ESM.pdf]

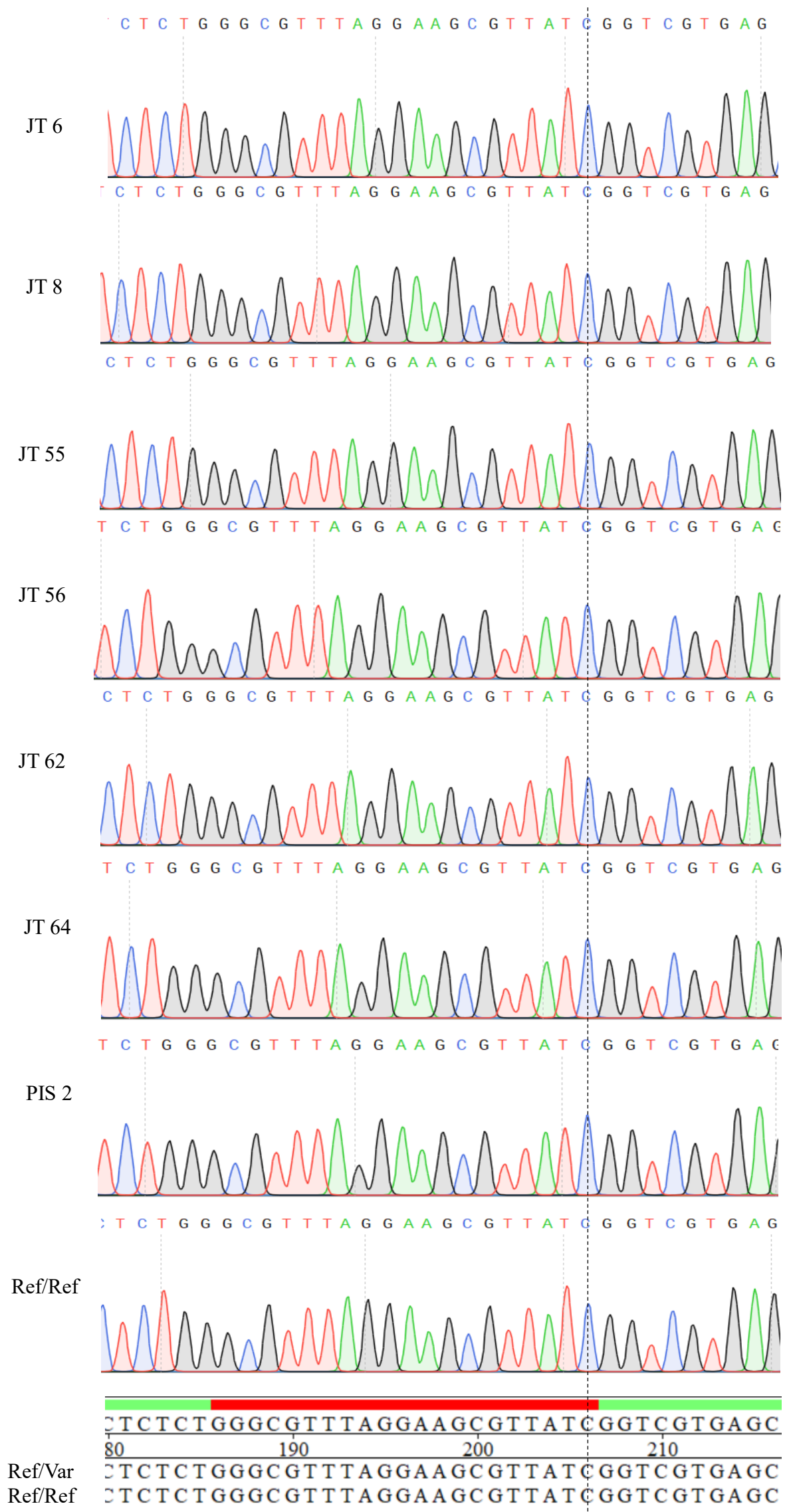

Figure S2. Validation of a mutation within the *ERG* gene through PCR and Sanger sequencing

Supplement: Supplementary file 5 — Additional file 5: Fig. S2. Validation of a mutation within the ERG gene through PCR and Sanger sequencing. [file 12864_2021_8089_MOESM5_ESM.pdf]
