# Supplementary material for: Genome-wide association study reveals 14 new SNPs and confirms two structural variants highly associated with the horned/polled phenotype in goats
Source: BMC Genomics. 2021 Oct 28;22:769. doi: 10.1186/s12864-021-08089-w (PMC8555091; doi:10.1186/s12864-021-08089-w)
Supplement: Supplementary file 8 — Additional file 8: Fig. S3. The validation of genetic sex for four PIS goats by PCR amplification and agarose gel electrophoresis. [file 12864_2021_8089_MOESM8_ESM.pdf]

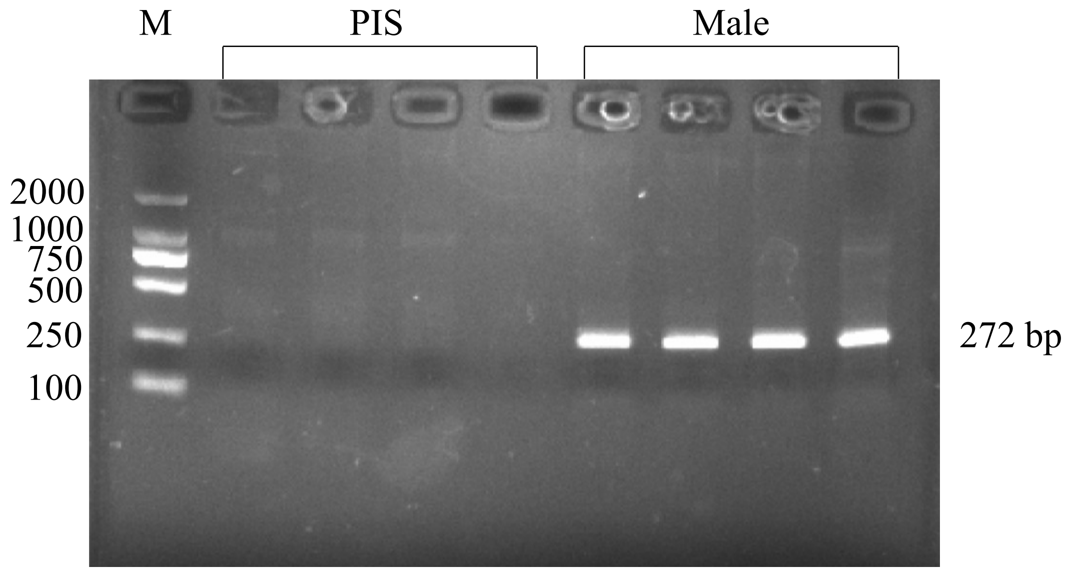

Figure S3. The validation of genetical sex for four PIS goats by PCR amplification and agarose gel electrophoresis
